# Glioblastoma with high O6-methyl-guanine DNA methyltransferase expression are more immunologically active than tumors with low *MGMT* expression

**DOI:** 10.3389/fimmu.2024.1328375

**Published:** 2024-01-15

**Authors:** Yoshihiro Kushihara, Shota Tanaka, Yukari Kobayashi, Koji Nagaoka, Miyu Kikuchi, Takahide Nejo, Erika Yamazawa, Shohei Nambu, Kazuha Kugasawa, Hirokazu Takami, Shunsaku Takayanagi, Nobuhito Saito, Kazuhiro Kakimi

**Affiliations:** ^1^ Department of Neurosurgery, Graduate School of Medicine, The University of Tokyo, Tokyo, Japan; ^2^ Department of Immunotherapeutics, The University of Tokyo Hospital, Tokyo, Japan; ^3^ Genome Science and Medicine, Research center for Advanced Science and technology, The University of Tokyo, Tokyo, Japan; ^4^ Department of Immunology, Kindai University Faculty of Medicine, Osakasayama, Osaka, Japan

**Keywords:** glioblastoma, O6-methyl-guanine DNA methyltransferase (*MGMT*), transcriptome, tumor-immune microenvironment, tumor-infiltrating lymphocyte

## Abstract

**Background:**

Glioblastoma (GBM) is a highly lethal brain tumor. The effectiveness of temozolomide (TMZ) treatment in GBM is linked to the methylation status of O6-methyl-guanine DNA methyltransferase (*MGMT*) promoter. Patients with unmethylated *MGMT* promoter have limited treatment options available. Consequently, there is a pressing need for alternative therapeutic strategies for such patients.

**Methods:**

Data, including transcriptomic and clinical information, as well as information on *MGMT* promoter methylation status in primary GBM, were obtained from The Cancer Genome Atlas (TCGA) (n=121) and Chinese Glioma Genome Atlas (CGGA) (n=83) datasets. Samples were categorized into high and low *MGMT* expression groups, MGMT-high (MGMT-H) and MGMT-low (MGMT-L) tumors. A comprehensive transcriptome analysis was conducted to explore the tumor-immune microenvironment. Furthermore, we integrated transcriptome data from 13 GBM patients operated at our institution with findings from tumor-infiltrating lymphocyte (TIL) cultures, specifically investigating their response to autologous tumors.

**Results:**

Gene signatures associated with various immune cells, including CD8 T cells, helper T cells, B cells, and macrophages, were noted in MGMT-H tumors. Pathway analysis confirmed the enrichment of immune cell-related pathways. Additionally, biological processes involved in the activation of monocytes and lymphocytes were observed in MGMT-H tumors. Furthermore, TIL culture experiments showed a greater presence of tumor-reactive T cells in MGMT-H tumors compared to MGMT-L tumors. These findings suggest that MGMT-H tumors has a potential for enhanced immune response against tumors mediated by CD8 T cells.

**Conclusion:**

Our study provides novel insights into the immune cell composition of MGMT-H tumors, which is characterized by the infiltration of type 1 helper T cells and activated B cells, and also the presence of tumor-reactive T cells evidenced by TIL culture. These findings contribute to a better understanding of the immune response in MGMT-H tumors, emphasizing their potential for immunotherapy. Further studies are warranted to investigate on the mechanisms of *MGMT* expression and antitumor immunity.

## 1 Introduction

Glioblastoma (GBM) is the most common and lethal malignant brain tumor. Despite its standard-of-care treatments, consisting of maximal safe surgical resection, radiotherapy and chemotherapy with temozolomide (TMZ), the median overall survival (OS) is approximately 16 months (1). It has been widely accepted that O6-methyl-guanine DNA methyltransferase (*MGMT*) promoter methylation in GBM is associated with a benefit from TMZ treatment (1, 2). The cytotoxic effects of TMZ are exerted by the induction of O6-methylguanine (O6mG), leading to the inhibition of DNA replication. MGMT is a DNA repair protein that removes the cytotoxic O6mG DNA lesions generated by TMZ; thereby, MGMT expression, which is suppressed by methylation of *MGMT* promoter, is mechanistically linked to TMZ resistance (3, 4). Patients with unmethylated *MGMT* promoter and high MGMT expression lack effective treatment options and have a poor prognosis. Therefore, there is an urgent need for a new treatment approach, especially for those patients.

Given the ongoing need for innovative treatment methods to enhance outcomes for glioblastoma patients and the proven effectiveness of immune checkpoint inhibitors (ICI) in different types of tumors, researchers are now exploring the potential of ICI in treating glioblastoma. However, thus far, all tested immunotherapies for glioblastoma (GBM) have been unsuccessful in improving clinical outcomes for unselected patient groups. Notably, trials using nivolumab (NIVO), an anti-PD-1 therapy, have failed to show a survival advantage in GBM patients. For instance, in the CheckMate 143 trial, NIVO did not outperform bevacizumab in unselected patients (5), and in the CheckMate 498 study, the combination of PD-1 blockade with radiotherapy did not improve survival compared to the cohort receiving temozolomide plus radiotherapy in patients with an unmethylated MGMT promoter (6). In another trial, CheckMate 548 found that NIVO, combined with temozolomide and radiotherapy, was not superior to temozolomide, radiotherapy, and placebo in newly diagnosed GBM patients with a methylated MGMT promoter (7). It is necessary to consider treatment options based on the characteristics of the intratumoral immune response in GBM.

A recent study analyzed the association between the main molecular profile of GBM and specific immunological markers (8). It found that the expression of CD8 and CD68, assessed by immunohistochemistry, was higher in GBM cases with unmethylated *MGMT* promoter than those with the methylated counterpart (9). This suggests that the difference in MGMT status contributes to the formation of a unique tumor microenvironment. The importance of *MGMT* methylation status is widely recognized and has been incorporated into clinical trials as well as decision-making for actual treatment for patients. However, among studies, various methodologies are leveraged, such as methylation-specific PCR (MSP), pyrosequencing, or more high-throughput genome-wide methylation arrays, which makes direct comparisons challenging. On the other hand, strong inverse correlations between *MGMT* methylation and its mRNA expression status have been reported. Therefore, in this study, we chose to focus on the transcript-level expression of *MGMT*, instead of its methylation status (4). Our study aimed to define further the immunological tumor microenvironment of GBM with low *MGMT* expression and elucidate its immunological features. We, for the first time, integrated transcriptome data with data from tumor-infiltrating lymphocyte (TIL) cultures to assess the actual contribution of the immunological tumor microenvironment.

## 2 Materials and methods

### 2.1 Patients

The discovery cohort for this study consisted of GBM data obtained from The Cancer Genome Atlas (TCGA). Transcriptomic and clinical data, along with information on *MGMT* promoter methylation status in primary GBM, were acquired from the TCGA Genome Data Commons Data portal (https://portal.gdc.cancer.gov) (download date; 2019/11/11) and cBioportal for Cancer Genomics (https://www.cbioportal.org)(download date; 2019/11/27), respectively. The TCGA-GBM dataset contained 155 cases of primary GBM, of which 121 cases had available information on *MGMT* promoter methylation. Consequently, the analysis was performed on this subset of 121 cases with known *MGMT* promoter methylation status.

The Chinese Glioma Genome Atlas (CGGA) dataset, specifically the mRNAseq_325 series, was employed as the validation cohort. Transcriptomic and clinical data, including information on *MGMT* promoter methylation status in primary GBM, were obtained from the CGGA database (http://www.cgga.org.cn/) (download date; 2019/09/09). Within the mRNAseq_325 series, a total of 85cases of primary GBM were identified, out of which 83 cases had available information on *MGMT* promoter methylation. Accordingly, the analysis focused on this subset.

Furthermore, an additional validation cohort, referred to as the University of Tokyo Hospital (UTH) cohort, was included in the analysis. This cohort comprised 13 consecutive primary GBM patients who underwent surgical resection at The University of Tokyo Hospital between November 2017 and December 2020. RNA samples were extracted from the resected tissues and subjected to RNA-sequencing (RNA-Seq) analysis. All procedures involving human participants were conducted in compliance with the institution’s ethical standards, following the guidelines outlined in the 1964 Helsinki Declaration and its subsequent revisions or comparable ethical standards. The study received approval from the research ethics committees of the University of Tokyo (Approval No. G3545), and written informed consent was obtained from all individual participants included in the study. Detailed patient characteristics for the three data cohorts are presented in **Table 1** and **Supplementary Table 1**.

**Table 1:** Patient characteristics of the three data cohorts.

		Discovery Cohort	Validation Cohort	Experimental Cohort	p
		TCGA_GBM Primary Tumor (n=121)	CGGA_mRNAseq325 GBM Primary Tumor (n=83)	TheUTH GBM Primary Tumor (n=13)	
Age at diagnosis, mean ± s.d.		60.8 ± 14.1	48.9 ± 12.3	63.3 ± 13.6	< 0.0001
Gender, n(%)	Male	73(60)	51(61)	7(54)	0.873
	Female	48(40)	32(39)	6(46)
MGMT promoter methylation, n(%)	Methylated	55(45)	32(39)	7(54)	0.454
	Unmethylated	66(55)	51(61)	6(46)
IDH1 mutation, n(%)	Wild type	113(93)	72(87)	13(100)	0.132
	Mutant type	8(7)	11(13)	0(0)

### 2.2 Clinical sample processing

GBM tumors were collected immediately after surgical resection and frozen in liquid nitrogen for subsequent RNA extraction. The tumor tissue was also processed using the Tumor Dissociation Kit, human (Miltenyi Biotec, Bergisch Gladbach, Germany) and the gentleMACS Octo Dissociator (Miltenyi) to ensure efficient dissociation. The resulting tissue suspensions were then filtered through a 70 μm filter. These suspensions, referred to as fresh tumor digest (FTD), were frozen and stored in a 1:1 mixture of CP-1 (Kyokuto Pharmaceutical Industrial Co. Ltd., Tokyo, Japan) and RPMI-1640 medium (Nacalai Tesque, Kyoto, Japan). FTD was stored in liquid nitrogen to maintain viability for future use in TIL culture.

### 2.3 RNA extraction

Total RNA samples from the fresh frozen tissues were extracted using the AllPrep DNA/RNA/miRNA Universal Kits (Qiagen, Hilden, Germany), following the manufacturer’s instructions. The extracted RNAs were then evaluated for quality and quantity. For next-generation sequencing (NGS), RNA samples meeting the following criteria were selected: a concentration of ≥ 20.0 ng/μL, a total amount of ≥ 0.4 μg, and a RNA integrity number (RIN) of ≥ 7.0, as assessed using the Agilent 2200 TapeStation (Agilent Technologies, Santa Clara, CA, USA).

### 2.4 RNA-sequencing (RNA-seq)

For RNA-Seq library preparation, the NEBNext^®^ UltraTM RNA Library Prep Kit for Illumina^®^ (Agilent Technologies) was utilized, following the manufacturer’s protocols. The prepared libraries were subjected to sequencing as 150-bp paired-end reads using the NovaSeq platform (Illumina, San Diego, CA, USA) at VERITAS (Danvers, MA, USA). Each sample yielded approximately 35.1 million reads of 150 base pairs in length on average. The obtained reads were then aligned to the reference genome (GRCh38/hg38) using STAR (v.2.5.2b) (10). Expression values were calculated as fragments per kilobase of exon per million fragments mapped (FPKM) using HTSeq (v.0.6.1) (11) and the R programming language (version 3.4.3; https://www.r-project.org/).

### 2.5 Differentially expressed genes (DEGs)

Samples were binarily classified into high and low expression groups, MGMT-high (MGMT-H) and MGMT-low (MGMT-L) tumors, according to the median value of *MGMT* mRNA expression. The raw counts obtained from RNA-Seq data were subjected to normalization. Subsequently, the differential expression analysis between MGMT-H tumors and MGMT-L tumors was performed using R version 3.6.2, utilizing the TCC (12) and edgeR (13) packages. Genes showing statistically significant differential expression were identified as differentially expressed genes (DEGs) based on the criteria of a p-value less than 0.05 and a False Discovery Rate (FDR) q-value less than 0.05.

### 2.6 Gene ontology (GO) functions enrichment analysis

We conducted a gene ontology (GO) functions enrichment analysis using Metascape (http://metascape.org) to elucidate the differences in the main activation processes associated with MGMT status. This comprehensive web resource facilitates data management and analysis (14). We obtained GO terms for the biological process (BP) category from the Molecular Signature Database v7.1 (MSigDB; https://www.gsea-msigdb.org/gsea/msigdb). The enrichment analysis of GO terms for biological processes was performed on the DEGs obtained from the TCC analysis using Metascape. Results were deemed significant if the p-value was less than 0.05 and the FDR q-value was less than 0.05.

### 2.7 Ingenuity pathway analysis (IPA)

DEGs obtained from the TCC analysis were analyzed using the Ingenuity Pathway Analysis (IPA) software (QIAGEN, Redwood City, CA, USA), accessible at https://www.qiagen.com/ingenuity. The core analysis in IPA encompassed various components, including canonical pathways, upstream regulators, regulator effects, and diseases and biological functions. Advanced algorithms incorporating machine learning techniques were utilized during the analysis (https://qiagen.my.salesforce-sites.com/KnowledgeBase/articles/Knowledge/Graphical-Summary). A Graphical Summary, consolidating the outcomes of the core analysis into a single network diagram, was generated to provide a concise representation of the results.

### 2.8 Gene set enrichment analysis (GSEA)

Gene set enrichment analysis (GSEA) was conducted to compare the expression levels between the MGMT-H and MGMT-L groups. Specifically, we employed GSEA version 4.1.0 to assess the differential expression of gene sets related to GO terms for BPs associated with characteristic functions in MGMT status. Additionally, we calculated a single-sample GSEA (ssGSEA) (15) score using R version 3.6.2 with the GSVA (16) package version 1.38.2. Results were deemed significant if the p-value was less than 0.05 and the FDR q-value was less than 0.05.

### 2.9 Calculation of tumor-infiltrating immune cell (TIC) fractions by transcriptome

To determine the proportions of tumor-infiltrating immune cell (TIC) fractions in MGMT-H and MGMT-L tumors, we employed CIBERSORTx and ssGSEA. For CIBERSORTx analysis, we utilized the absolute-mode algorithm based on the LM22 gene signature. The LM22 gene signature was obtained from https://CIBERSORTx.stanford.edu/. The algorithm was executed with 1000 permutations to estimate the proportions of TICs. This allowed us to quantify specific immune cell types within the tumor microenvironment. In parallel, we performed ssGSEA (15) using R version 3.6.2 with the GSVA (16) package version 1.38.2. The ssGSEA analysis was conducted using 28 subpopulations of TILs gene sets (17), referred to as “Charoentong_TIL_28 immunophenotype” in this study. This method enabled the assessment of the enrichment scores for each TIC subpopulation, providing insights into the immune landscape of the tumors. Furthermore, we calculated the “Tumor Immune and Dysfunction and Exclusion (TIDE) score,” “Dysfunction” score, and “Exclusion” scores using the TIDE web application (http://tide.dfci.harvard.edu/) (18). These scores measure tumor immune response, immune dysfunction, and immune exclusion, respectively.

### 2.10 Hierarchical clustering

We utilized an unsupervised hierarchical clustering algorithm for the transcriptome analysis data, which included GO terms BP process ssGSEA scores and TIC fractions. This analysis used R version 3.6.2 with the pheatmap package version 1.0.12. To generate the hierarchical clustering, we calculated the squared Euclidean distance between the samples. This distance measure quantifies the dissimilarity between samples based on their transcriptome profiles. We then applied an agglomerative algorithm with Ward’s method, which iteratively merges clusters to minimize the within-cluster variance.

### 2.11 Molecular diagnosis

Regarding the IDH mutations observed in GBM, they were identified using the Sanger method. Polymerase chain reaction (PCR) was performed using tumor DNA from 13 cases in the UTH cohort. For IDH1 mutations, KOD FX Neo (Toyobo, Osaka, Japan) DNA polymerase was utilized, while for IDH2 mutations, AmpliTaq GoldTM DNA Polymerase with Buffer I (Applied Biosystems, Waltham, MA) was employed. **Supplementary Table 2A** presents the primer sequences, annealing temperatures, and lengths of the amplified PCR fragments for IDH mutation analysis. Sequence analysis of the PCR product was performed by FASMAC Corporation (Kanagawa, Japan). Mutation analysis was performed with DNADynamo software (BLUE TRACTOR SOFTWARE Ltd, North Wales, UK).

For the assessment of *MGMT* promoter methylation, MSP was employed. Tumor DNA was subjected to bisulfite conversion using the EZ DNA Methylation-Gold Kit (Zymo Research, Irvine, CA) following the provided protocol. The primers were designed to amplify the CpG-rich region of the *MGMT* promoter region based on a previous publication (19). **Supplementary Table 2B** provides the primer sequences, annealing temperatures, and lengths of the resulting PCR fragments for *MGMT* promoter methylation analysis. Following PCR, electrophoresis was performed to determine the presence or absence of methylation in the *MGMT* promoter region. Episcope^®^ Methylated GCT116 gDNA (Takara Bio Inc., Shiga, Japan) was used as a methylation control, and Episcope^®^ Unmethylated GCT116 DKO gDNA (Takara Bio Inc., Shiga, Japan) was used as an unmethylated control for methylation determination.

### 2.12 Immunohistochemistry

Immunohistochemistry (IHC) was performed on 4μm-thick sections prepared from formalin-fixed paraffin-embedded (FFPE) samples. Automated IHC staining was conducted at Kyodo Byori Co., Ltd. (Kobe, Japan), using specific antibodies diluted with BOND Polymer Refine Detection (Leica Biosystems, Newcastle, UK) on the Leica Bond-MAX automated immunohistochemistry staining system, following the manufacturer’s instructions. The antibodies used were targeted against CD4, CD8, CD20, CD68, and CD163. Each section was digitally imaged using the BIOREVO-9000 fluorescence microscope (Keyence, Osaka, Japan), and the BZ-II Analyzer image analysis software (Keyence, Osaka, Japan) was utilized to quantify the area of IHC positive staining and calculate the IHC positive staining area per unit tumor area (μm2).

### 2.13 TIL culture

Under sterile conditions, surgically resected tumor specimens from the UTH cohort were divided into three parts: one for RNA-Seq, one for FTD and one for TIL culture. For TIL culture, tumors were minced using scalpels immediately after resection. The minced tumor tissues were then incubated for 2-3 weeks at 37°C in RPMI 1640 medium (Nacalai Tesque) supplemented with CTS™ Immune Cell Serum Replacement (5%, Gibco, NY, USA), HEPES buffer solution (10mM, Dojindo, Kumamoto, Japan), MEM Non-essential Amino Acids Solution (Wako, Osaka, Japan), Sodium Pyruvate (1mM, Wako, Osaka, Japan), 2-mercaptoethanol (Invitrogen, CA, USA), penicillin/streptomycin (Nacalai Tesque), Interleukin-2 (IL-2) (6000U/mL, PeproTech, NJ, USA), and an Indoleamine 2,3-dioxygenase inhibitor (IDOi) called 1-methyl-L-tryptophan (100uM, Sigma-Aldrich, MO, USA). The tissue and culture medium were placed in a 24-well plate (Corning, Corning, NY). The cultivation period for TIL was set to 2-3week. The lymphocyte count in the TIL culture medium was determined using flow cytometry. Live cells were identified with 7-AAD Viability Staining Solution (BioLegend, #420404), and mononuclear cells within that subset were gated and counted using flow-count beads, Flow-Count Fluorospheres (Beckman Coulter, #7547053). Stained cells were analyzed on a Gallios flow cytometer (Beckman Coulter), and data were processed using Kaluza (Beckman Coulter). Positive TIL proliferation was defined as obtaining 3.0×10^5^ or more TILs per well (**Supplementary Figure 1A**). The TIL culture rate was calculated as the ratio of the number of wells with positive TIL proliferation to the total number of cultured wells. This measure assessed TIL culture’s success rate in terms of obtaining viable and proliferating TILs.

### 2.14 Interferonγ (IFNγ) Enzyme-Linked Immuno-Sorbent Assay (ELISA)

To aassess the tumor reactivity of cultured TIL, FTD was thawed and examined for the viability of the tumor cells. Only FTD with satisfactory viability of tumor cells was utilized. Subsequently, the FTD was co-cultured with TIL for 20-24 hours. TIL and FTD were also independently cultured for 20-24 hours as background controls. At the time of thawing, FTD was evaluated for viability. After incubation, the culture supernatant was collected, and the levels of IFNγ were measured using an ELISA kit (Thermo Fisher Scientific, Waltham, MA, USA) following the manufacturer’s protocols.

The tumor-reactive IFNγ was calculated using the following formula:


Tumor-reactive IFNγ=IFNγ (TIL+FTD)−[IFNγ (TIL)+IFNγ (FTD)]

Here, IFNγ (TIL+FTD) represents the amount of IFNγ in the supernatant of the TIL+FTD co-culture, IFNγ (TIL) represents the amount of IFNγ in the supernatant of TIL alone, and IFNγ (FTD) represents the amount of IFNγ in the supernatant of FTD alone.

The tumor-specific immune response was considered positive if the amount of tumor-reactive IFNγ exceeded 100 pg/ml (**Supplementary Figure 1B**). Each patient’s tumor-reactive immune response rate was defined as the ratio of the number of wells exhibiting a tumor-reactive immune response to the total number of cultured wells.

### 2.15 Statistics

The statistical analyses for continuous variables were performed with the Wilcoxon rank-sum test. In the comparison of three groups for continuous variables, the Kruskal-Wallis test was performed. The analyses for nominal variables were performed with Fisher’s exact test. Statistical significance was set at P < 0.05 and FDR < 0.05 except for the differential gene expression and gene set enrichment analysis. All statistical analyses and plotting were performed using R 3.6.2. or JMP Pro 16 (SAS Institute Japan, Tokyo, Japan).

## 3 Results

### 3.1 DEGs in MGMT-H and MGMT-L tumors

**Figure 1:**
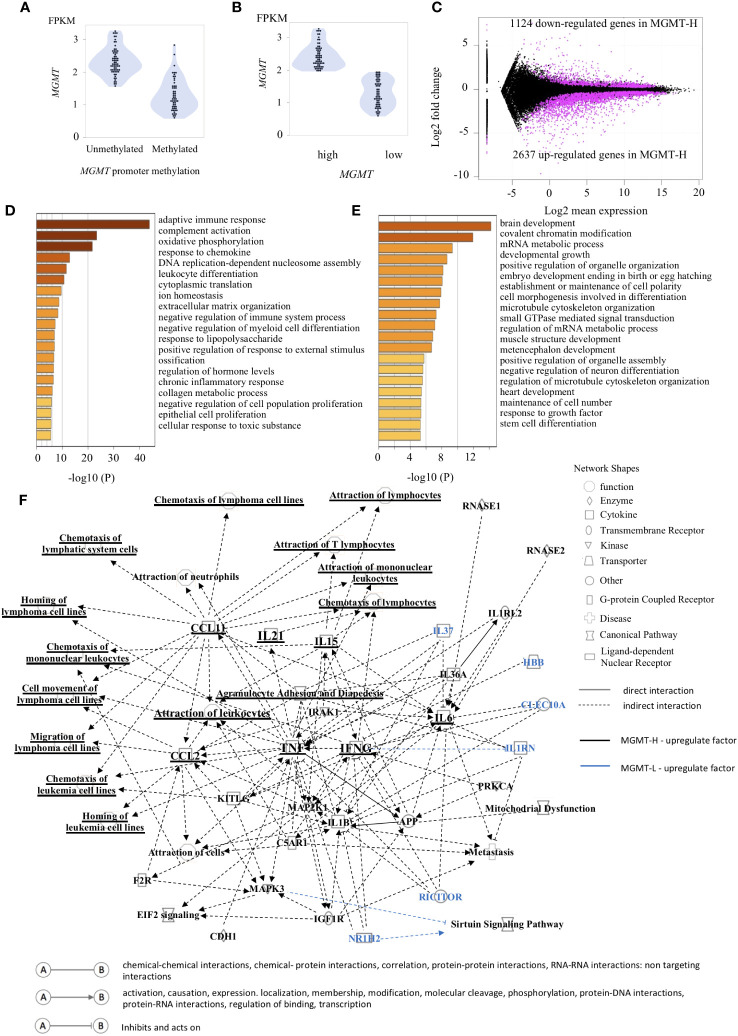
Gene expression patterns characteristic of MGMT-H/L groups. **(A)** The relationship between the methylation of the *MGMT* promoter region and gene transcription is depicted in panel. **(B)** The samples were classified into two groups, namely low expression and high expression, based on the median value of *MGMT* expression. **(C)** The M-A plot illustrates the differential expression of genes (DEGs) that are either up-regulated or down-regulated in the TCGA GBM dataset. This plot shows the relationship between the average concentration (log mean expression) and fold-change (log fold change) across the genes. Genes with dots located above 0 on the y-axis indicate lower expression in MGMT-H patients compared to MGMT-L patients, while genes with dots located below 0 on the y-axis indicate higher expression in MGMT-H patients compared to MGMT-L patients. Each gene is represented by a black dot on the plot. The magenta dots indicate significant DEGs that meet the criteria for a significant adjusted P value of less than 0.05 and an FDR q-value of less than 0.05. A Metascape enrichment analysis was conducted to identify statistically enriched ontology terms (specifically, Gene Ontology Biological Process terms) using the set of DEGs. A bar graph was generated to display the enriched terms associated with the upregulated gene set in MGMT-H **(D)** and MGMT-L **(E)**. Each term is represented by a bar, and the color of each bar corresponds to the p-value associated with the enrichment. The color gradient reflects the significance of the enrichment, with darker shades indicating more significant p-values. **(F)** The Ingenuity Pathway Analysis (IPA) was utilized to generate graphical pathways based on the DEGs. In the graphical pathways, factors expected to be highly expressed in MGMT-H are represented by a black legend, while factors expected to be highly expressed in MGMT-L are represented by a blue legend. The network analysis revealed that genes related to adaptive immune reactions, such as IFNG and TNF, were highly upregulated in MGMT-H. Consequently, this led to an upregulation of lymphocyte chemotaxis in MGMT-H. In contrast, genes such as IL37, RICTOR, NR1H2 were highly expressed in MGMT-L.

The *MGMT* gene is epigenetically silenced through its promoter methylation, leading to decreased MGMT expression. However, factors other than *MGMT* promoter methylation, such as p53, SP-1, and NF-κB, are also known to regulate MGMT expression (3). Interestingly, some cases with *MGMT* promoter methylation exhibit high MGMT expression (**Figure 1A**). Given the lack of standardized methodology for methylation analysis (3), we classified the samples into two groups based on *MGMT* mRNA expression levels: MGMT-H (high expression) and MGMT-L (low expression). The classification used the median value of *MGMT* mRNA expression as the threshold (**Figure 1B**; **Supplementary Table 1**). This binary classification approach allows us to compare the characteristics and outcomes between the high and low *MGMT* expression groups across different cohorts.

In our comprehensive gene expression analysis of the TCGA-GBM cohort, we compared the RNA-seq data of MGMT-H and MGMT-L tumors. Our analysis revealed 3761 DEGs between them. Among these DEGs, 2637 were up-regulated, and 1124 were down-regulated in MGMT-H tumors (**Figure 1C**). We performed pathway and process enrichment analysis using the GO Biological Process in the Metascape database to gain insights into the biological functions associated with these DEGs. The DEGs up-regulated in MGMT-H tumors were found to be closely related to immune response processes, including “adaptive immune response,” “complement activation,” and “response to chemokine” (**Figure 1D**). On the other hand, the DEGs up-regulated in MGMT-L tumors were primarily involved in gene replication, expression, and regulation processes, such as “brain development,” “covalent chromatin modification,” and “mRNA metabolic process” (**Figure 1E**). Furthermore, we conducted IPA to gain further insights into the underlying mechanisms and downstream effects of the observed gene expression changes (**Figure 1F**). The IPA analysis indicated that factors such as IFNG, TNF, IL21, CCL2, and CCL11 are expected to be up-regulated in the MGMT-H group. This suggests enhanced lymphocyte migration through activating these factors in MGMT-H tumors. Our findings highlight the distinct biological functions and pathways associated with MGMT-H and MGMT-L tumors, particularly in immune response and gene regulation processes.

### 3.2 GSEA analysis of MGMT-H and MGMT-L tumors

In the Metascape analysis, each DEG’s gene expression levels were not considered. To conduct a more comprehensive analysis, we performed GSEA, which incorporates gene expression levels. GSEA analysis was conducted on MGMT-H and MGMT-L tumors using the MSigDB Biological Process category. Among the top 30 activated processes in MGMT-H tumors (**Supplementary Table 3A**), immune-related processes were predominant. Conversely, the top 30 activated processes in MGMT-L tumors were primarily associated with gene replication, expression, and regulation (**Supplementary Table 3B**).

**Figure 2:**
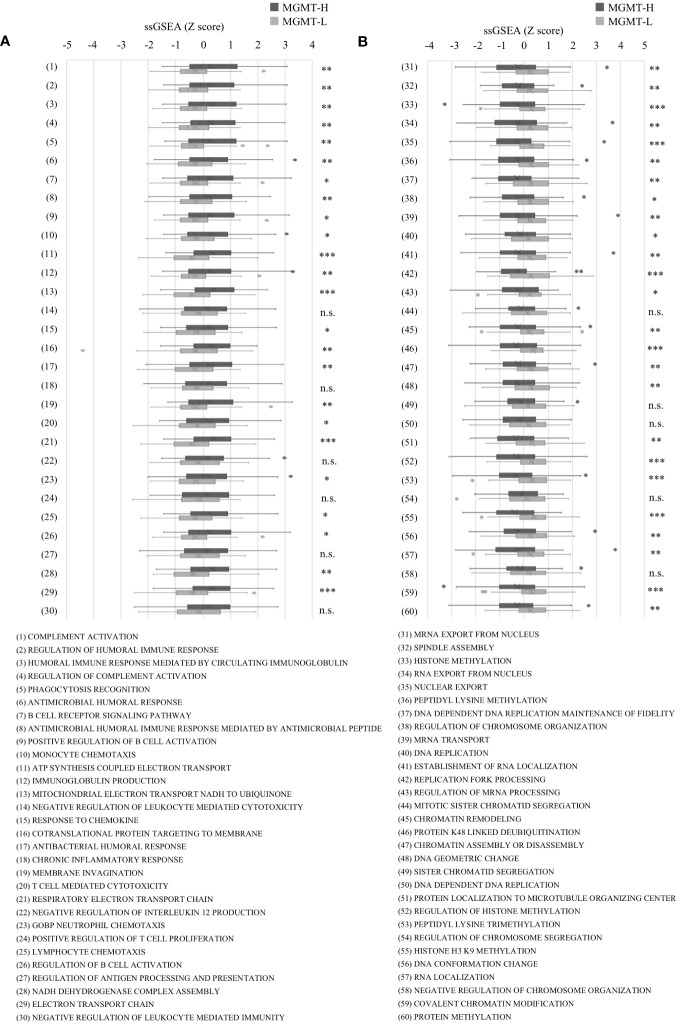
The ssGSEA values in the top 30 most highly expressed gene sets in each of the MGMT-H/L groups in MSigDB C5 Biological Process gene sets. ssGSEA was performed using gene sets associated with the top 30 most highly expressed genes in MGMT-H **(A)** and MGMT-L **(B)** tumors. Wilcoxon test, *: P <0.05; **: P <0.01; ***: P <0.001; n.s.: P ≧0.05.

To further investigate these top 30 biological processes, we performed ssGSEA on each patient. The ssGSEA scores of these biological processes were compared between MGMT-H and MGMT-L tumors (**Figure 2**). T cell-related immune processes, such as “T cell-mediated cytotoxicity” and “lymphocyte chemotaxis,” were found to be activated in MGMT-H tumors (**Figure 2A**). These findings suggest that MGMT-H tumors exhibit a more potent anti-tumor immunity induction against GBM cells than MGMT-L tumors. Additionally, B cell-related immune processes, including “complement activation,” “regulation of humoral immune response,” “positive regulation of B cell activation,” and “regulation of complement activation,” were also activated in MGMT-H tumors. These results indicate a potential connection between B cell immunity and anti-tumor immunity in MGMT-H tumors or suggest the formation of tertiary lymphoid structure in the tumor. Furthermore, the process of monocyte migration (“monocyte chemotaxis”) and the process associated with antigen recognition for phagocytosis by macrophages and antigen-presenting cells (“phagocytosis recognition”) were activated. These results suggest the activation of T cell-mediated anti-tumor immunity in MGMT-H tumors. In line with the Metascape analysis, immune-related processes were not found to be activated in MGMT-L tumors. Instead, processes associated with GBM tumor characteristics, such as cell division, gene expression, and histone modification, were found to be activated (**Figure 2B**).

### 3.3 Immune cell profiling and phenotyping of MGMT-H and MGMT-L tumors

**Figure 3:**
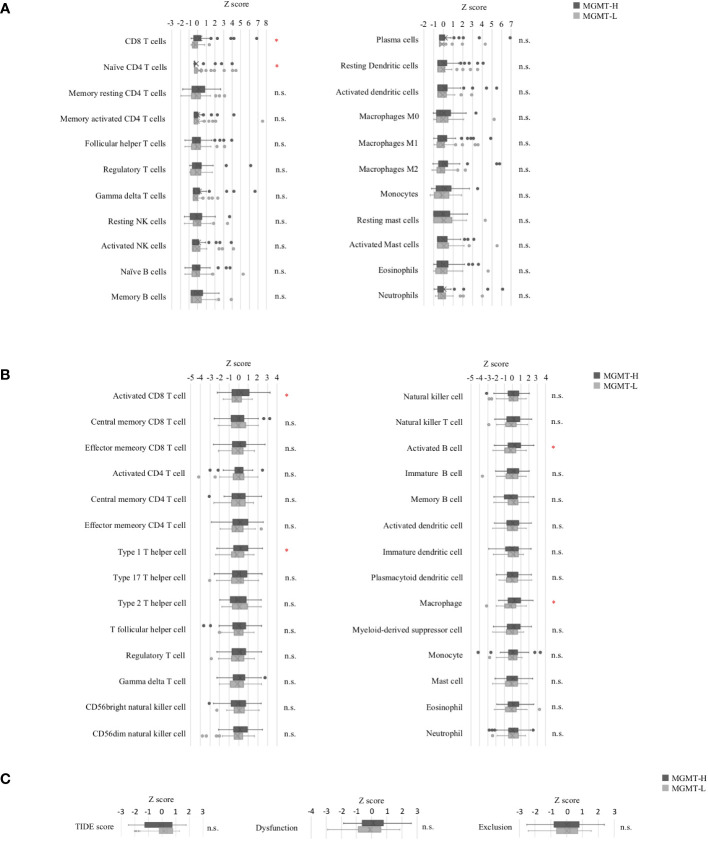
Tumor-infiltrating immune cells in MGMT-H/L tumors. **(A)** The levels of immune cell infiltration were compared between MGMT-H and MGMT-L tumors using CIBERSORTx-Absolute scores. **(B)** The immunophenotypes in MGMT-H/L tumors were compared by ssGSEA using gene sets reported by Charoentong et al. (17) **(C)** The Tumor Immune Dysfunction and Exclusion (TIDE) analysis method was employed to assess the parameters of TIDE, Dysfunction, and Exclusion in the two groups, MGMT-H and MGMT-L. Wilcoxon test, *: *P <*0.05; n.s.: *P* ≧0.05.

Subsequently, we conducted immune cell profiling to quantify the abundance and identify the specific types of immune cells infiltrating the tumors. We utilized the LM22 signature matrix within the CIBERSORTx platform for this analysis, which covers 22 immune cell types (**Figure 3A**). However, it is important to note that the LM22 immune subsets do not provide information regarding the phenotype, activation, or differentiation status of the immune cells. To overcome this limitation, we implemented the ssGSEA method using a set of 28 subpopulations of TILs gene sets, referred to as the “Charoentong TIL 28 immunophenotype” (17) (**Figure 3B**). This approach allowed us to examine T cell phenotypes and functional states more comprehensively by incorporating these gene sets. By leveraging these specific gene sets, we gained insights into T cells’ phenotypic and functional characteristics within the tumor microenvironment. This methodology provides a more detailed understanding of the diverse T cell populations and their functional states in the tumors under investigation.

In the CIBERSORTx analysis, we observed that the scores for CD8 T cells were significantly higher (p = 0.015), while the scores for naive CD4 T cells were significantly lower (p = 0.028) in MGMT-H tumors compared to MGMT-L tumors (**Figure 3A**). However, there were no significant differences in other immune cell populations between MGMT-H and MGMT-L tumors. Furthermore, when utilizing the Charoentong 28 TIL immunophenotype gene set analysis, we found that the ssGSEA scores for activated CD8 T cells (p = 0.040), type 1 T helper cells (p = 0.026), activated B cells (p = 0.015), and macrophages (p = 0.017) were significantly higher in MGMT-H tumors compared to MGMT-L tumors (**Figure 3B**). These findings reinforce the notion that immune responses are actively engaged and enhanced in MGMT-H tumors. Taken together, these results support the notion that MGMT-H tumors exhibit heightened immune activation and potentially more robust anti-tumor immune responses compared to MGMT-L tumors.

Furthermore, we utilized the TIDE web application (http://tide.dfci.harvard.edu) (18) to assess immune evasion signatures (**Figure 3C**). The dysfunction scores, which reflect the degree of T cell dysfunction, were slightly higher in MGMT-H tumors compared to MGMT-L tumors, although the difference did not reach statistical significance (p = 0.405). These findings indicate that MGMT-H tumors exhibit a higher level of T cell infiltration that may have undergone functional impairment or dysfunction.

### 3.4 Validation with the CGGA GBM cohort

**Figure 4:**
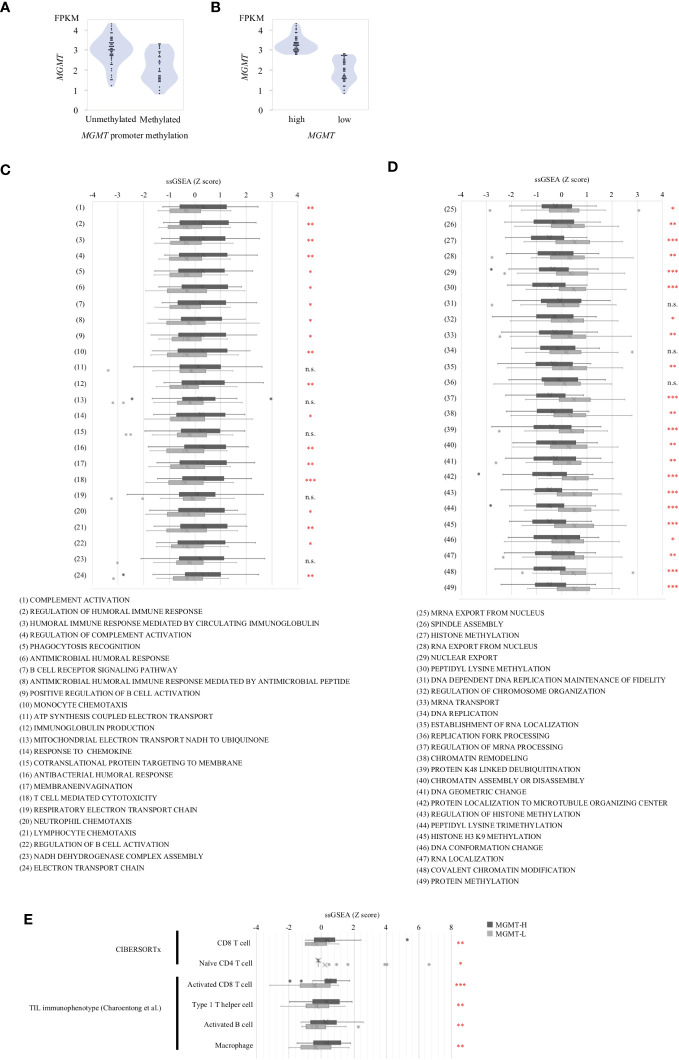
Validation Analysis in CGGA cohort. **(A)** *MGMT* mRNA expression in CGGA human GBM correlated with the methylation of *MGMT* promoter region. **(B)** The samples were classified into two groups, namely low expression and high expression, based on the median value of *MGMT* expression. Gene sets from the MSigDB Biological Process category that significantly different between MGMT-H and MGMT-L tumors in TCGA cohort were applied to CGGA cohort. **(C)** The set of genes within MSigDB C5 BP that were highly expressed in the MGMT-H group detected in the TCGA database were validated in CGGA. **(D)** The set of genes within MSigDB C5 BP that were highly expressed in the MGMT-L group detected in the TCGA database were validated in CGGA. **(E)** Immunophenotypes highly expressed in the MGMT-H group detected in the TCGA database were validated in CGGA. Wilcoxon test, *: P <0.05; **: P <0.01; ***: P <0.001; n.s.: P ≧0.05.

We extended our analysis to validate the pathways enriched in MGMT-H or MGMT-L tumors and the highly expressed infiltrating immune cell phenotypes using the CGGA GBM cohort. We observed that *MGMT* expression was generally higher in *MGMT* promoter unmethylated tumors compared to methylated tumors. However, it is worth noting that some *MGMT* promoter methylated tumors still exhibited high levels of *MGMT* expression (**Figure 4A**). Therefore, similar to the discovery cohort, we classified samples into low and high groups based on the median value of *MGMT* expression (**Figure 4B**).

Using the MSigDB C5 BP gene sets that are highly expressed in each of the MGMT-H/L groups identified in the comparison between the MGMT-H/L groups in the TCGA GBM cohort, we also performed ssGSEA analysis in the CGGA GBM cohort. ssGSEA values of those gene sets were used to compare the two MGMT-H/L groups in the CGGA GBM cohort (**Figures 4C, D**). Consistent with the findings in the TCGA cohort, 19 out of 24 gene sets enriched in MGMT-H tumors displayed higher ssGSEA scores in MGMT-H tumors compared to MGMT-L tumors in the CGGA GBM cohort (**Figure 4C**). Similarly, 22 out of 25 gene sets enriched in MGMT-L tumors showed higher ssGSEA scores in MGMT-L tumors compared to MGMT-H tumors (**Figure 4D**). Furthermore, Digital cytometry results in the TCGA GBM cohort were also validated in the CGGA GBM cohort. Specifically, we calculated the absolute scores of T cell CD8 and T cell CD4 naive using CIBERSORTx and the ssGSEA scores of activated CD8 T cell, type 1 T helper cell, activated B cell, and macrophage using the Charoentong TIL 28 immunophenotype gene set. These values were used for MGMT-H/L intergroup comparisons (**Figure 4E**). Notably, we obtained similar results in the CGGA GBM cohort, further supporting the consistency of our findings across different datasets. Overall, the validation in the CGGA GBM cohort provides robustness to our results, confirming the enriched pathways and immune cell phenotypes characteristic of MGMT-H and MGMT-L tumors identified in the discovery cohort.

### 3.5 Immunophenotyping of GBM using selected gene sets

**Figure 5:**
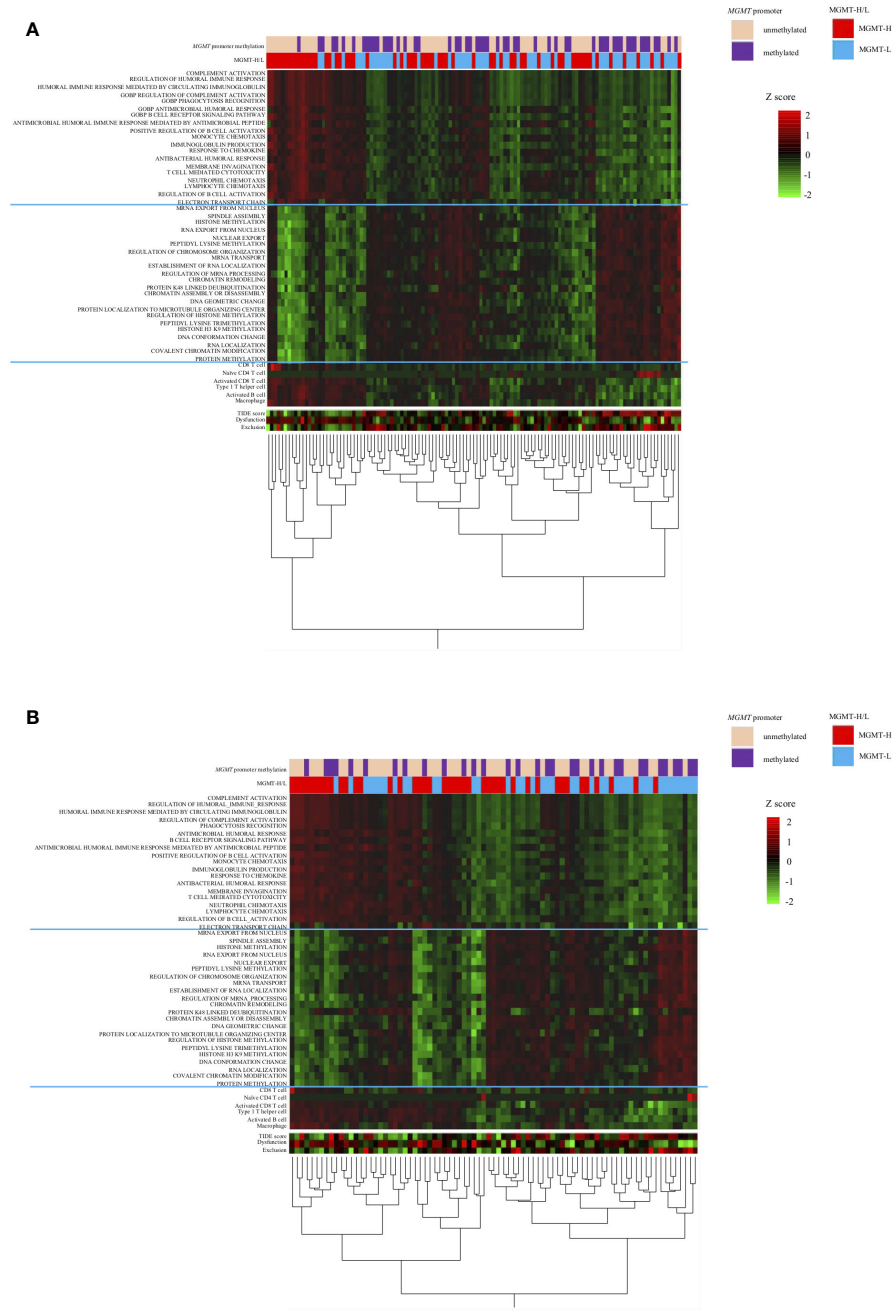
Heat map analysis using factors that have been selected from the TCGA-GBM and CGGA cohorts’ analysis. **(A)** TCGA primary GBM cohort. **(B)** CGGA primary GBM cohort.

The distinct gene sets are summarized in **Table 2** that characterize MGMT-H and MGMT-L GBM tumors by analyzing the TCGA cohort and validating the findings using the CGGA cohort. Based on the gene sets enriched in MGMT-H and MGMT-L tumors, we immunophenotyped the GBM tumor microenvironment through hierarchical clustering (**Figure 5**). In **Figure 5A**, we observed a subgroup of cases with higher scores for immune-related gene sets, including activated CD8 T cell, type 1 T helper cell, activated B cell, and macrophage. The TIDE and Dysfunction scores were high in these cases, while the Exclusion scores were low. These results suggest an increased immune response and infiltration of immune cells into the tumor microenvironment in these cases. Conversely, there was another subgroup of cases with higher scores for GBM tumor-related processes, such as cell division, gene expression, and histone modification. These results indicate a dominance of tumor-specific processes in these cases. Consistent with the TCGA cohort, we observed a similar pattern in the CGGA GBM cohort (**Figure 5B**). Immune-related gene sets were more activated in MGMT-H tumors compared to MGMT-L tumors, while GBM tumor-related process gene sets were more activated in MGMT-L tumors compared to MGMT-H tumors. Overall, these findings demonstrate the distinct immunophenotypes and gene expression profiles associated with MGMT-H and MGMT-L GBM tumors, highlighting the complex interplay between the tumor microenvironment and tumor-specific processes.

**Table 2:** The distinct gene sets that characterize MGMT-H and MGMT-L GBM tumors.

	High expression gene sets in MGMT-H	High expression gene sets in MGMT-L
MSigDB C5 Biological Process	COMPLEMENT ACTIVATION REGULATION OF HUMORAL IMMUNE RESPONSE HUMORAL IMMUNE RESPONSE MEDIATED BY CIRCULATING IMMUNOGLOBULIN REGULATION OF COMPLEMENT ACTIVATION PHAGOCYTOSIS RECOGNITION ANTIMICROBIAL HUMORAL RESPONSE B CELL RECEPTOR SIGNALING PATHWAY ANTIMICROBIAL HUMORAL IMMUNE RESPONSE MEDIATED BY ANTIMICROBIAL PEPTIDE POSITIVE REGULATION OF B CELL ACTIVATION MONOCYTE CHEMOTAXIS IMMUNOGLOBULIN PRODUCTION RESPONSE TO CHEMOKINE ANTIBACTERIAL HUMORAL RESPONSE MEMBRANE INVAGINATION T CELL MEDIATED CYTOTOXICITY NEUTROPHIL CHEMOTAXIS LYMPHOCYTE CHEMOTAXIS REGULATION OF B CELL ACTIVATION GOBP_ELECTRON_TRANSPORT_CHAIN	MRNA EXPORT FROM NUCLEUS SPINDLE ASSEMBLY HISTONE METHYLATION RNA EXPORT FROM NUCLEUS NUCLEAR EXPORT PEPTIDYL LYSINE METHYLATION REGULATION OF CHROMOSOME ORGANIZATION MRNA TRANSPORT ESTABLISHMENT OF RNA LOCALIZATION REGULATION OF MRNA PROCESSING CHROMATIN REMODELING GPROTEIN K48 LINKED DEUBIQUITINATION CHROMATIN ASSEMBLY OR DISASSEMBLY DNA GEOMETRIC CHANGE PROTEIN LOCALIZATION TO MICROTUBULE ORGANIZING CENTER REGULATION OF HISTONE METHYLATION PEPTIDYL LYSINE TRIMETHYLATION HISTONE H3 K9 METHYLATION DNA CONFORMATION CHANGE RNA LOCALIZATION COVALENT CHROMATIN MODIFICATION PROTEIN METHYLATION
CIBERSORTx	CD8 T cell	Naïve CD4 T cell
Charoentong TIL 28 immunophenotype	Activated CD8 T cell Type 1 T helper cell Activated B cell Macrophage	

### 3.6 Molecular diagnosis and immunohistochemical analysis

The results of the analysis in the TCGA and CGGA cohorts were also validated in the UTH cohort.

**Figure 6:**
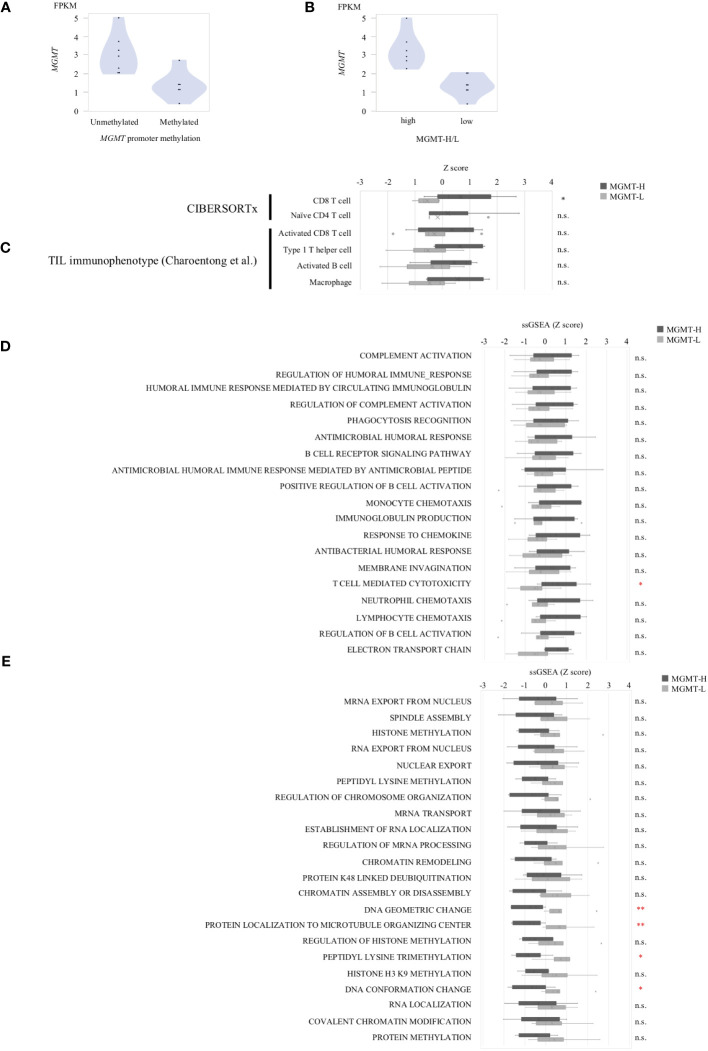
The gene expression profiles of MGMT-H/L tumors in UTH dataset. **(A)** *MGMT* mRNA expression in human GBM correlated with the methylation of *MGMT* promoter region. **(B)** Samples were divided into two groups, low and high, according to *MGMT* median expression level. **(C)** CIBERSORTx and ssGSEA analysis using gene sets that were significantly different in the TCGA-GBM cohort. ssGSEA value were compared between MGMT-H/L tumors using MSigDB C5 Biological Process Gene sets that were significantly different between MGMT-H **(D)** and MGMT-L **(E)** in the TCGA-GBM cohort. Wilcoxon test, *: *P <*0.05; **: *P <*0.01; n.s.: *P* ≧0.05.

**Figure 7:**
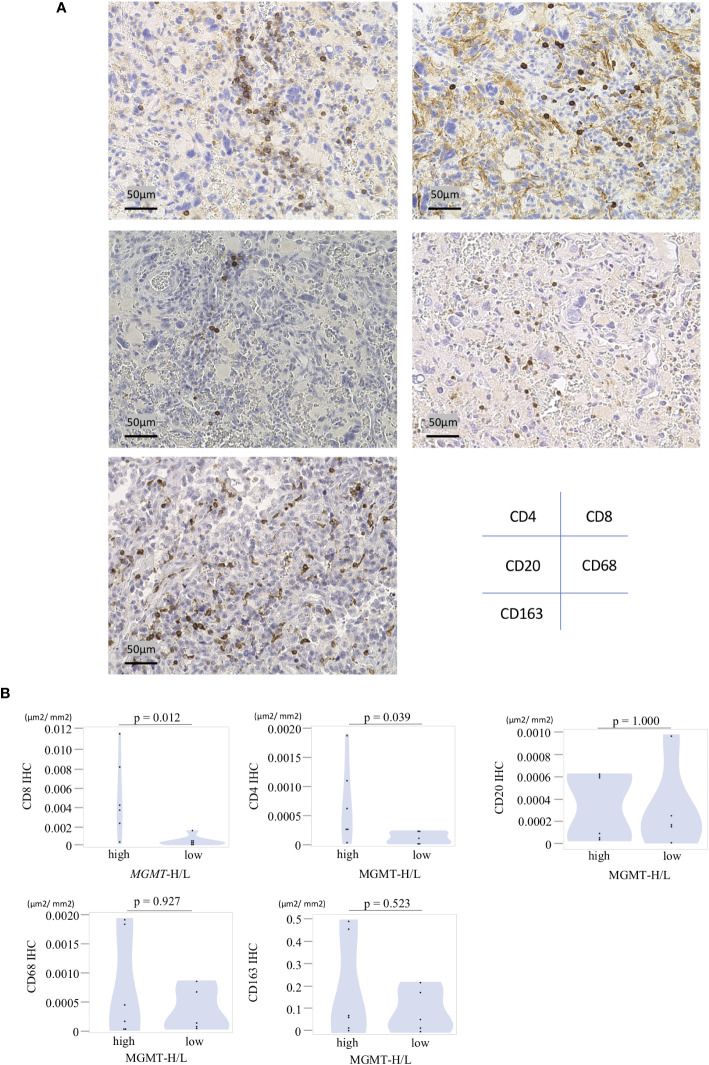
Immunohistochemical analysis of MGMT-H/L tumors. **(A)** FFPE slides were subjected to immunostaining for CD4^+^, CD8^+^, CD20^+^, CD68^+^, and CD163^+^ cells within the tumor. Representative examples of each marker were presented at a magnification of x200. **(B)**The area of positive signals was automatically measured by the BIOREVO-9000 fluorescence microscope (Keyence, Osaka, Japan), and the BZ-II Analyzer image analysis software (Keyence) was utilized to quantify the area of IHC positive staining and calculate the IHC positive staining area per unit tumor area(μm2). The ratio of positive cell area to GBM tumor area was calculated and compared between the MGMT-H/L groups.

In the UTH cohort, consisting of 13 GBM patients, all cases were IDH wild-type, with *MGMT* promoter methylation observed in 6 cases and unmethylation in 7cases (**Supplementary Table 4**). We divided them into MGMT-H group (6 patients) and MGMT-L group (7 patients) based on their *MGMT* expression levels (**Figures 6A, B**). First, the results of the digital cytometry analysis were also validated in the UTH cohort. Consistent with the findings in the TCGA cohort, the CIBERSORTx analysis revealed a higher abundance of CD8 T cells in MGMT-H tumors compared to MGMT-L tumors (p = 0.015) (**Figure 6C**). Otherwise, there were no significant differences between MGMT-H/L groups in naive CD4 T cell, activated CD8 T cell, type 1 helper T cell, activated B cell, and macrophage. However, in activated CD8 T cell, type 1 helper T cell, activated B cell, and macrophage, numbers tended to be higher in the MGMT-H group. Validation was then performed on the results of the gene set in the MSigDB C5 Biological Process. Although statistical significance was not reached, GSEA analysis also showed a trend of more activated immunological phenotypes in MGMT-H tumors (**Figures 6D, E**). To further investigate the infiltration of immune cells into the tumors, we performed immunohistochemical analysis on FFPE tissues from the UTH cohort. Specifically, we examined the presence of CD4^+^, CD8^+^, CD20^+^, CD68^+^, and CD163^+^ cells within the tumor microenvironment (**Figure 7A**; **Supplementary Table 4**). As anticipated, the immunohistochemical analysis revealed a higher infiltration of CD8^+^ (p = 0.012) and CD4^+^ (p = 0.039) cells in MGMT-H tumors compared to MGMT-L tumors (**Figure 7B**). No significant difference was observed between the two groups for CD20, CD68, and CD163 (p = 1.000, p = 0.927, p = 0.523). These results from the UTH cohort corroborate the findings from the TCGA cohort, indicating a consistent pattern of increased infiltration of CD8^+^ and CD4^+^ cells in MGMT-H tumors. These results suggest a potential association between *MGMT* expression levels and the immune cell composition within the tumor microenvironment.

### 3.7 Tumor-specific immune response

**Figure 8:**
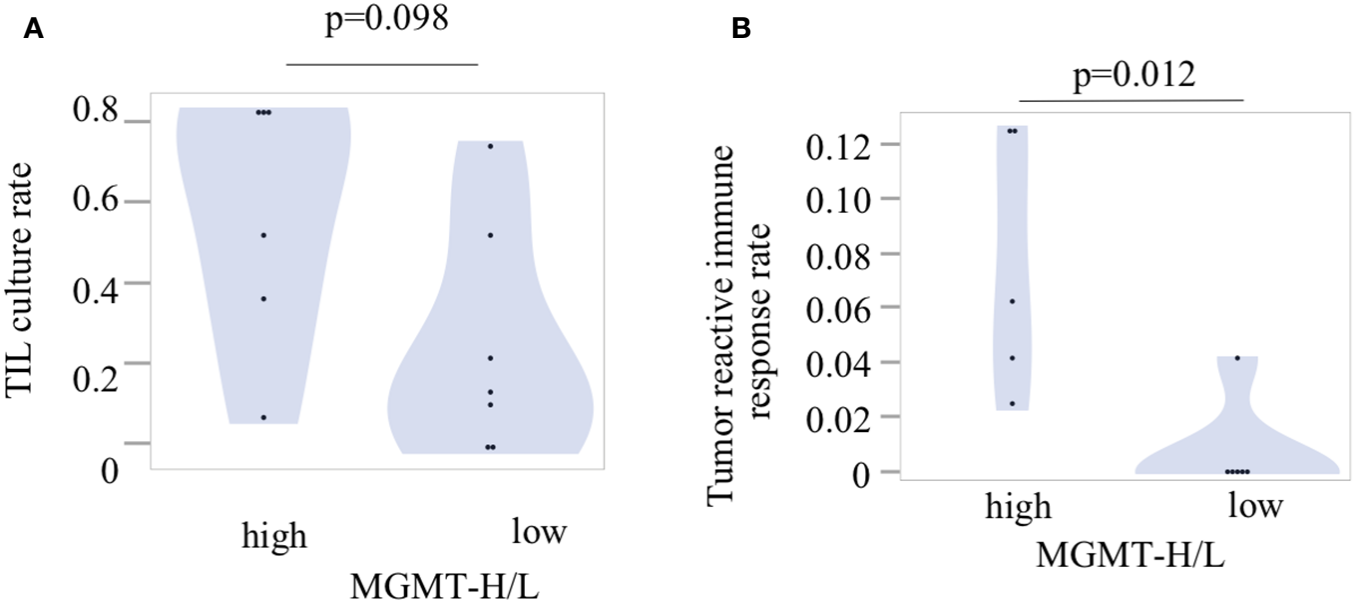
Tumor-infiltrating lymphocytes and their reactivity to the tumors. **(A)** The TIL culture rate was calculated as the ratio of the number of wells with positive TIL proliferation to the total number of cultured wells. **(B)** The tumor reactivity of cultured TILs was determined by IFNγ production after incubation of TILs and fresh tumor digest (FTD). The culture supernatant was collected, and the levels of IFNγ were measured using an ELISA. Each patient’s tumor-reactive immune response rate was defined as the ratio of the number of wells exhibiting a tumor-reactive immune response to the total number of cultured wells.

To investigate tumor-specific T cells within the tumors, we conducted TIL culture experiments in UTH cohort. Tumor samples were finely minced into small 2-3 mm pieces using a surgical scalpel and then cultured with IL-2 for 2 to 3 weeks in a 24-well plate. The proliferation of TILs was observed in 11 out of 13 cases, with 6 out of 6 MGMT-H tumors and 5 out of 7 MGMT-L tumors showing successful TIL expansion. From a total of 407 wells used for TIL cultures, we achieved the expansion of TILs to reach a cell count of 3×10^5^ or more per well in 150 wells (**Supplementary Table 4**). Consequently, the overall TIL culture rate was determined to be 36.9%. When considering the MGMT-H and MGMT-L tumors separately, the TIL culture rate was 47.5% and 26.1%, respectively (**Figure 8A**). However, the difference between these two groups did not reach statistical significance (p=0.098).

Following the expansion of TILs, we examined their reactivity to autologous tumors by assessing their production of IFNγ during co-culture with tumors cryopreserved as FTD (**Supplementary Table 4**). Out of the 150 wells with TIL proliferation, co-culture experiments with tumors could not be conducted in 6 wells from 2 cases due to insufficient cryopreserved tumor specimens. Therefore, co-culturing with the tumor was performed in 144 wells, including 94 wells from 5 cases of MGMT-H tumors and 50 wells from 4 cases of MGMT-L tumors (**Supplementary Table 4**). The concentration of IFNγ in the culture supernatant was measured using ELISA, and wells exhibiting IFNγ levels of 100 pg/ml or higher were considered to indicate a tumor-specific immune response. We observed the production of IFNγ in 12 wells from 6 cases, including 11 wells from 5 cases of MGMT-H tumors and 1 well from 1 case of MGMT-L tumor (**Supplementary Table 4**). These results demonstrate that tumor-specific immune responses were significantly higher in MGMT-H tumors than in MGMT-L tumors (p = 0.012) (**Figure 8B**).

**Figure 9:**
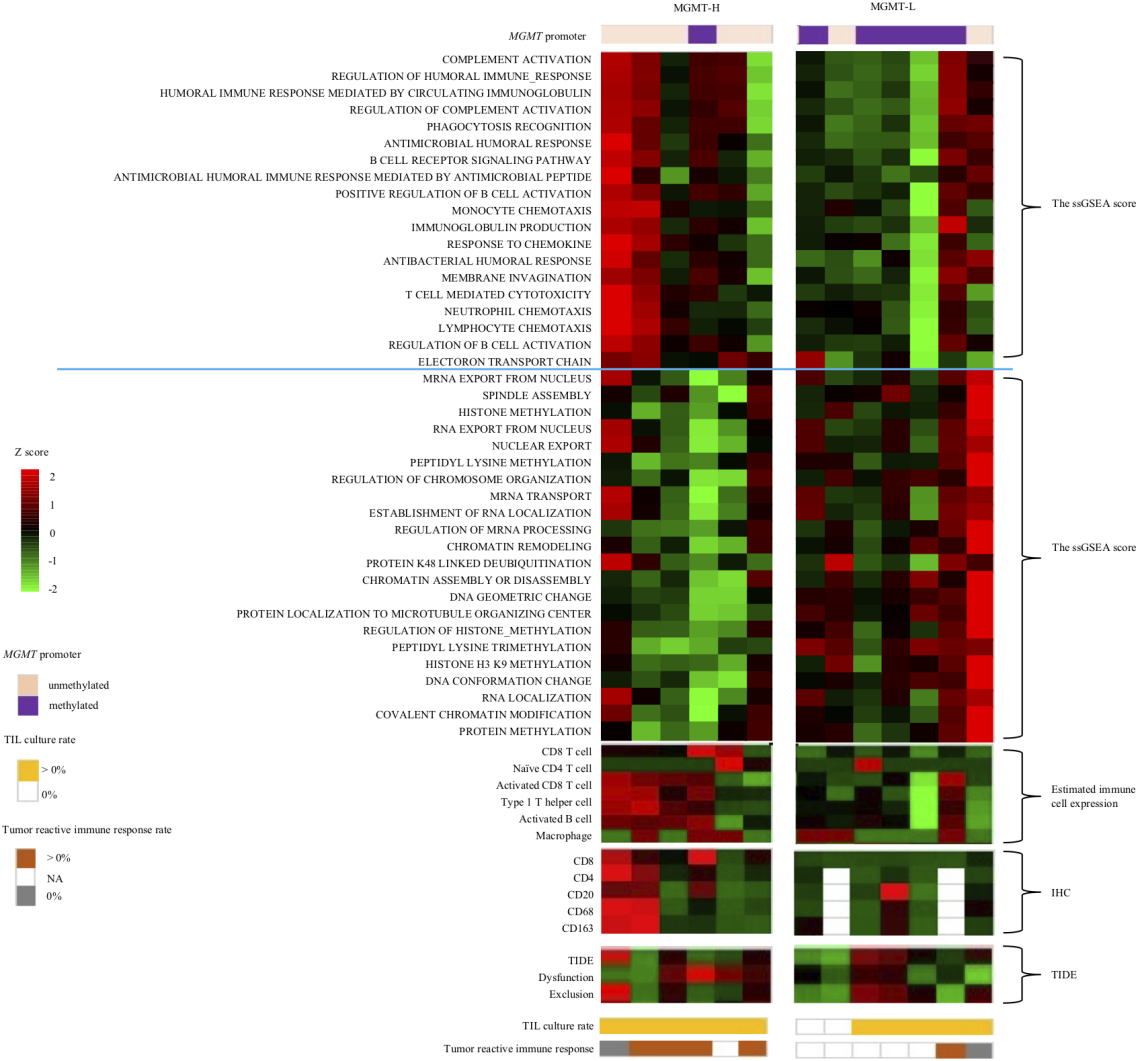
Integrated analysis of tumor microenvironment of MGMT-H/L GBM. In the UTH cohort, a heat map analysis using factors that have been selected from the TCGA-GBM and CGGA cohorts’ analysis, IHC result and TIL culture result. Patients in the MGMT-H group had higher expression of immune-related genes and higher expression of activated CD8 T cell, type 1 helper cell and activated B cell. In addition, such patients tended to have a higher incidence of tumor reactive immune response.

To provide a comprehensive view of the findings, we integrated the transcriptome data, immunohistochemical analysis, and TIL culture data into a heat map comparing MGMT-H and MGMT-L tumors (**Figure 9**). The heat map illustrates the co-expression of activated CD8 T cells, type 1 helper cells, activated B cells, and macrophages in specific cases within the MGMT-H tumors. Notably, these MGMT-H tumors also tended to elicit a tumor-specific immune response.

In summary, our study revealed that MGMT-H tumors displayed activation of adaptive immunity, particularly involving CD8 cells and type 1 helper T cells, which contributed to the induction of a tumor-specific immune response. These findings highlight the importance of understanding the immunological landscape of MGMT-H tumors and suggest potential targets for immunotherapy interventions to enhance tumor-specific immune responses in GBM.

## 4 Discussion

Understanding the interaction between the tumor and the immune system is crucial for developing effective treatments for GBM, particularly for patients with an unmethylated MGMT promoter and high MGMT expression, who face limited treatment options and a poor prognosis. This study investigated the relationship between MGMT expression or MGMT promoter methylation and tumor immunity. Consistent with a recent analysis of GBM’s molecular profile and specific immunological markers, which revealed higher expression of CD8 and CD68 in GBM cases with an unmethylated MGMT promoter compared to the methylated counterpart (9), our comprehensive genetic analysis consistently demonstrated enhanced immune responses in GBM with MGMT-H tumors. This was evident through the up-regulation of gene signatures associated with tumor-infiltrating immune cells. Significantly, TIL culture experiments indicated a greater presence of tumor-reactive T cells in MGMT-H tumors compared to MGMT-L tumors. These findings suggest that MGMT-H tumors have the potential for antitumor immune responses mediated by CD8 T cells.

Based on our study results, **Supplementary Figure 2** presents a schematic diagram illustrating the expected tumor immune status in MGMT-H/L, respectively. Our study contributes to the field in two novel aspects. Firstly, we demonstrate for the first time that MGMT-H tumors exhibit a more significant infiltration of type 1 helper T cells and activated B cells. These immune cell subtypes are crucial in orchestrating effective immune responses against tumors (20–22). Identifying these cell types in MGMT-H tumors adds to our understanding of the immune landscape and highlights potential targets for immunotherapeutic interventions. Secondly, our *in vitro* TIL culture experiments provide novel insights by demonstrating that MGMT-H tumors harbor more tumor-reactive T cells. This observation extends beyond the mere abundance of T cells in MGMT-H tumors and confirms the functional reactivity of the existing T cells toward the tumor. Our results were consistent with the previous report that the combination of neoantigen quality and T lymphocyte infiltrates was associated with the longest survival of GBM patients (23).These findings hold significant implications for developing immunotherapies tailored to exploit the existing immune response in MGMT-H tumors.

One notable finding in this study is the up-regulated signature of activated B cells detected in MGMT-H tumors (**Figure 3B**; **Table 2**). Antigen presentation is critical in activating naïve CD8 T cells, and antigen-presenting cells, including B cells, are instrumental in this process (24). The emerging research has highlighted the involvement of B cells in antigen presentation within the tumor microenvironment (22). Furthermore, the presence of tertiary lymphoid structures (TLS) has been identified within tumors, including GBM (25, 26). TLS is an organized immune cell structure that resembles to secondary lymphoid organs and contributes to local immune responses. TLS formation has been associated with improved responsiveness to immunotherapy in various cancer types, such as melanoma (27). Considering these findings, the increased signature of activated B cells in MGMT-H tumors suggests their potential role in antigen presentation and the formation of TLS within the tumor microenvironment. Zhou et al. stratified glioma into three distinct tumor subtypes with the gene expression profile of TLS genes (28). The C subtype glioma with high immune infiltration was poor prognosis without immune checkpoint blockade therapy. These findings may have implications for understanding the immune response and potential immunotherapeutic strategies in GBM. Further research is needed to investigate the precise mechanisms and functional significance of activated B cells and TLS in MGMT-H tumors and their potential impact on the efficacy of immunotherapy.

Despite CD8 T cells showing activation of anti-tumor immunity in MGMT-H tumors, previous studies have indicated that the achieved immune response is insufficient to control the growth of GBM based on clinical data (1, 29). Past reports indicate that even in cases presenting MGMT-H with MGMT-unmethylated status, efficacy with Nivo alone cannot be anticipated (6). It is speculated that MGMT-H tumors may contain immunosuppressive factors that hinder the cytotoxicity of CD8 T cells. One such factor is the presence of highly expressed macrophages in MGMT-H tumors, known as tumor-associated macrophages (TAMs) (30–32). TAMs have different functional classifications, with anti-inflammatory TAMs being predominant in GBM (33). These anti-inflammatory TAMs suppress T cell function and pro-inflammatory TAM activities, contributing to the immunosuppressive microenvironment (33, 34). Targeting anti-inflammatory TAMs is a reasonable strategy to modulate the immunosuppressive environment and enhance the therapeutic effect and CSF-1R may be one such example. Inhibiting CSF-1R signaling can reduce anti-inflammatory TAMs and promote a pro-inflammatory phenotype, improving anti-tumor immune responses (30, 31). However, further research is needed to determine the safety, efficacy, and optimal treatment combinations for CSF-1R-targeted therapy in GBM. The complex tumor microenvironment and interactions between immune cell populations present challenges in developing effective immunotherapies. Nonetheless, targeting TAMs may hold promise for immunotherapy in GBM.

To clarify the relationship between *MGMT* expression or *MGMT* promoter methylation and tumor immunity, further investigations are needed. One approach could be creating an orthotopic murine model by injecting GBM cell lines with MGMT knockout or overexpression. This model would allow quantification of intratumoral immune cell infiltration, for example, by assessing TIL expression levels through techniques such as flow cytometry, IHC or RNA-Seq. By comparing the degree of *MGMT* expression or promoter methylation, with the level of immune cell infiltration, we can gain insights into the association between *MGMT* and tumor immunity.

This study has several limitations that should be acknowledged. Firstly, the cases included in the experiment were obtained from a single institution, resulting in a relatively small sample size. Including a larger number of cases from multiple institutions in future studies is imperative. Secondly, the transcriptome analysis conducted in this study focused on tumor bulk samples, limiting the ability to analyze individual immune cells’ specific functions and interactions. Although TAMs originate from brain-resident microglia and blood-derived monocytes, deconvolution of immune cells from bulk RNA-Seq data cannot discriminate between microglia and monocytes, nor can it identify astrocytes that are enriched in GBM with microglia. Incorporating single-cell analysis techniques would be valuable in evaluating the detailed expression levels and functions of each immune cell. Thirdly, the immunohistochemical staining method employed in this study only targeted specific markers, such as CD8 T cells. Multi-color analysis for type 1 helper T cells, activated B cells, and macrophages are necessary. Furthermore, analyzing the three-dimensional spatial relationship between these immune cells within the tumor microenvironment would provide insights into their cell-cell interactions. Lastly, the analysis in this study was limited to transcriptome analysis, and it is important to supplement the findings with whole exome sequencing data and methylome analysis. This will allow us to explore the relationship between MGMT status and factors such as neoantigens, gene mutations, and methylation patterns. Addressing these limitations in future studies will provide a more comprehensive understanding of the relationship between *MGMT* and the immune landscape in GBM.

## 5 Conclusions

Our study presents novel findings by characterizing the immune cell composition of MGMT-H tumors, highlighting the infiltration of activated CD8 T cells, type 1 helper T cells, activated B cells, and macrophages and revealing the presence of tumor-reactive T cells by TIL culture experiments. These results offer valuable insights into future immunotherapeutic strategies specifically targeting MGMT-H tumors.

## Data availability statement

The raw RNA-Seq data were deposited in the DNA Data Bank of Japan (DDBJ) under the accession number DRA016557. The original contributions presented in the study are included in the article/**Supplementary Material**, further inquiries can be directed to the corresponding author.

## Ethics statement

The studies involving humans were approved by the research ethics committees of the University of Tokyo. The studies were conducted in accordance with the local legislation and institutional requirements. The participants provided their written informed consent to participate in this study.

## Author contributions

YKu: Conceptualization, Data curation, Investigation, Visualization, Writing – original draft. STan: Conceptualization, Investigation, Writing – original draft. YKo: Data curation, Investigation, Methodology, Writing – review & editing. KN: Data curation, Investigation, Methodology, Writing – review & editing. MK: Investigation, Writing – review & editing. TN: Investigation, Writing – review & editing. EY: Investigation, Writing – review & editing. SN: Investigation, Writing – review & editing. KKu: Investigation, Writing – review & editing. HT: Investigation, Writing – review & editing. STak: Investigation, Writing – review & editing. NS: Investigation, Supervision, Writing – review & editing. KKa: Conceptualization, Funding acquisition, Project administration, Supervision, Validation, Writing – original draft.
