# Supplementary material for: Glioblastoma with high O6-methyl-guanine DNA methyltransferase expression are more immunologically active than tumors with low MGMT expression
Source: Front Immunol. 2024 Jan 15;15:1328375. doi: 10.3389/fimmu.2024.1328375 (PMC10824125; doi:10.3389/fimmu.2024.1328375)
Supplement: Supplementary file 1 [file DataSheet_1.pdf]

Supplementary Material

Glioblastoma with high O6-methyl-guanine DNA methyltransferase (*MGMT*) expression are more immunologically active than tumors with low *MGMT* expression

Yoshihiro Kushihara[1,2], Shota Tanaka[1], Yukari Kobayashi[2], Koji Nagaoka[2], Miyu Kikuchi[1], Takahide Nejo[1], Erika Yamazawa[1,3], Shohei Nambu[1], Kazuha Kugasawa[1], Hirokazu Takami[1], Shunsaku Takayanagi[1], Nobuhito Saito[1], and Kazuhiro Kakimi[2,4*]

[1]Department of Neurosurgery, Graduate School of Medicine, The University of Tokyo, Bunkyo-Ku, Tokyo 113-8655, Japan.

[2]Department of Immunotherapeutics, The University of Tokyo Hospital, Bunkyo-Ku, Tokyo 113-8655, Japan.

[3]Genome Science and Medicine, Research center for Advanced Science and technology, The University of Tokyo, Meguro-Ku, Tokyo 153-8904, Japan.

[4]Department of Immunology, Kinki University Faculty of Medicine, Osakasayama, Osaka 589-8511, Japan

*** Correspondence:**
Corresponding Author: Kazuhiro Kakimi, MD, PhD
kakimi@med.kindai.ac.jp

1 Supplementary Figures and Tables

1.1 Supplementary Figures

Supplementary Figure 1. Scatter plots depicting TIL proliferation and the increased IFNγ levels in TIL during co-culture with FTD in the UTH cohort.

A. TIL proliferation in the UTH cohort

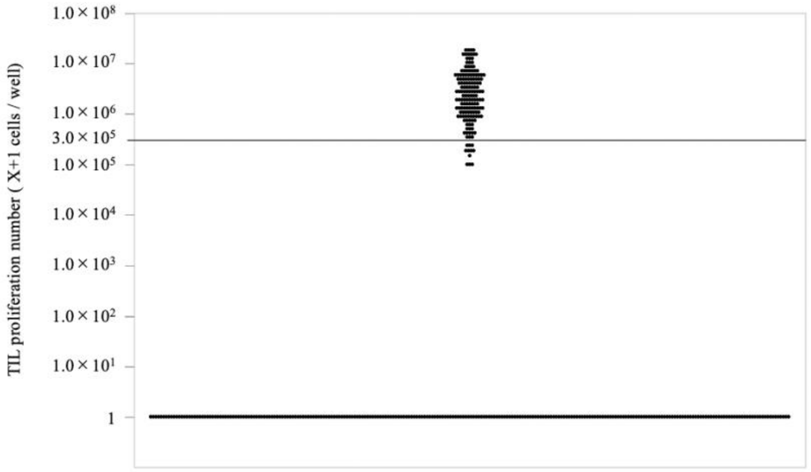

B. Increased IFNγ levels in TIL during co-culture with FTD in the UTH cc

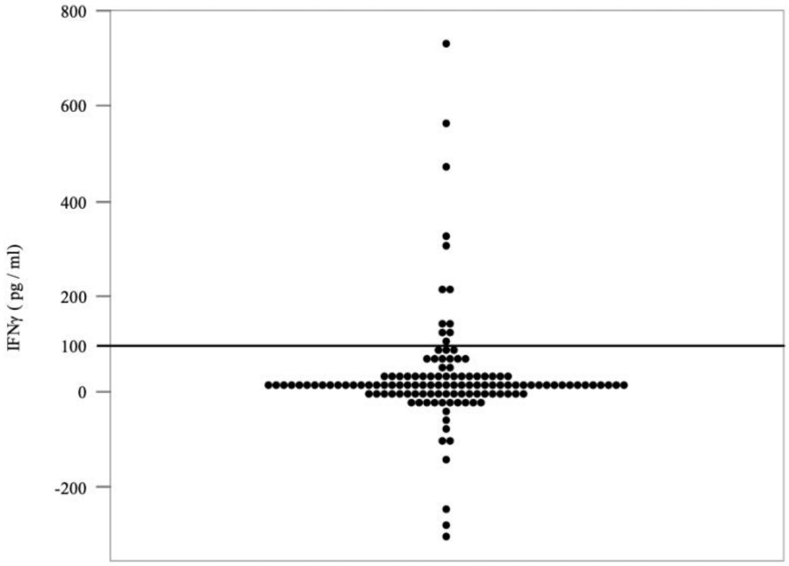

Supplementary Figure 2. Schematic illustrating differences in immune environment between MGMT-H/L Groups.

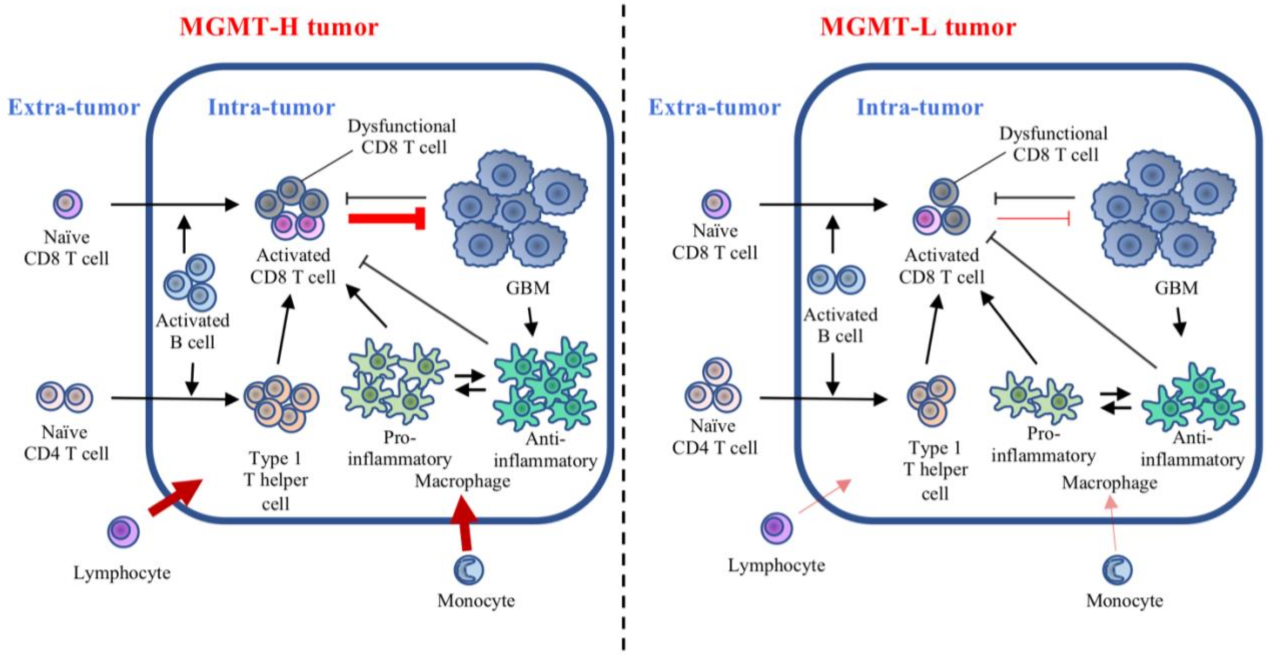

1.2 Supplementary Tables

Supplementary Table 1. Comparison between the MGMT-H group and MGMT-L group within each cohort, including TCGA GBM, CGGA (mRNAseq_325) GBM, and the UTH cohort.

		Discovery Cohort			Validation Cohort			Experimental Cohort		
		TCGA_GBM Primary Tumor (n=121)			CGGA_mRNAseq325 GBM Primary Tumor (n=83)			The UTH GBM Primary Tumor (n=13)		
		MGMT-H	MGMT-L	p	MGMT-H	MGMT-L	p	MGMT-H	MGMT-L	p
Age at diagnosis, mean±s.d.		63.5±13.5	59±13.8	0.049	49.0±13.6	48.9±11.2	0.967	68.3±13.2	59.0±13.3	0.283
Gender, n(%)	Male	39(64)	34(57)	0.460	28(68)	23(55)	0.261	4(67)	3(43)	0.592
	Female	22(36)	26(43)		13(32)	19(45)		2(33)	4(57)	
MGMT promoter methylation, n(%)	Methylated	6(10)	49(82)	<0.0001	8(20)	24(57)	0.0006	1(17)	5(71)	0.102
	Unmethylated	55(90)	11(18)		33(80)	18(43)		5(83)	2(29)	
IDH1 mutation, n(%)	Wild type	60(98)	53(88)	0.032	38(93)	34(81)	0.194	6(100)	7(100)	-
	Mutant type	1(2)	7(12)		3(7)	8(19)		0(0)	0(0)	

The p value was calculated using Wilcoxon rank sum or Fisher exact tests. CGGA: Chinese Glioma Genome Atlas, GBM: Glioblastoma, IDH1: isocitrate dehydrogenase1, MGMT: O6-methyl-guanine DNA methyltransferase, s.d.: standard deviation, TCGA: The Cancer Genome Atlas

Supplementary Table 2. Analysis conditions for molecular diagnosis in the UTH cohort.

A. Analysis conditions for IDH mutations.

Primer	Forward primer sequence	Reverse primer sequence	Annealing temperature (°C)	Estimated size of PCR product (bp)
IDH1	TGCCACCAACGACCAAGTCA	GCAAAATCACATTATTGCCAAC	59	295
IDH2	CTCTGTCCTCACAGAGTTCAAGC	CCACTCCTTGACACCACTGCC	59	345

B. Analysis conditions for methylation of the *MGMT* promoter region.

Primer	Forward primer sequence (5'-3')	Reverse primer sequence (5'-3')	Annealing temperature (°C)	Estimated size of PCR product (bp)
Methylated	TTTCGACGTTCGTAGGTTTTCGC	GCACTCTTCCGAAAACGAAACG	59	81
Unmethylated	TTTGTGTTTTGATGTTTGTAGGTT TTTGT	AACTCCACACTCTTCCAAAAACAAAA CA	59	93

Supplementary Table 3. Association between MGMT-H/L Status and MSigDB Gene Ontology C5 Biological Process in Primary GBM Patients.

A. Top 30 gene set enriched in MGMT-H

NAME	NES	NOM p	FDR q
COMPLEMENT ACTIVATION	3.374	<0.001	<0.001
REGULATION OF HUMORAL IMMUNE_RESPONSE	3.373	<0.001	<0.001
HUMORAL IMMUNE RESPONSE MEDIATED BY CIRCULATING IMMUNOGLOBULIN	3.314	<0.001	<0.001
REGULATION OF COMPLEMENT ACTIVATION	3.250	<0.001	<0.001
PHAGOCYTOSIS RECOGNITION	2.983	<0.001	<0.001
ANTIMICROBIAL HUMORAL RESPONSE	2.913	<0.001	<0.001
B CELL RECEPTOR SIGNALING PATHWAY	2.720	<0.001	<0.001
ANTIMICROBIAL HUMORAL IMMUNE_RESPONSE MEDIATED BY ANTIMICROBIAL PEPTIDE	2.704	<0.001	<0.001
POSITIVE REGULATION OF B CELL ACTIVATION	2.580	<0.001	<0.001
MONOCYTE CHEMOTAXIS	2.575	<0.001	<0.001
ATP SYNTHESIS COUPLED ELECTRON TRANSPORT	2.565	<0.001	<0.001
IMMUNOGLOBULIN PRODUCTION	2.556	<0.001	<0.001
MITOCHONDRIAL ELECTRON TRANSPORT NADH TO UBIQUINONE	2.523	<0.001	<0.001
NEGATIVE REGULATION OF LEUKOCYTE MEDIATED CYTOTOXICITY	2.426	<0.001	<0.001
RESPONSE TO CHEMOKINE	2.394	<0.001	0.001
COTRANSLATIONAL PROTEIN TARGETING TO MEMBRANE	2.355	<0.001	0.001
ANTIBACTERIAL HUMORAL RESPONSE	2.335	<0.001	0.001
CHRONIC INFLAMMATORY RESPONSE	2.334	0.007	0.001
MEMBRANE INVAGINATION	2.330	<0.001	0.001
T CELL MEDIATED CYTOTOXICITY	2.321	<0.001	0.001
RESPIRATORY ELECTRON TRANSPORT CHAIN	2.318	<0.001	0.001
NEGATIVE REGULATION OF INTERLEUKIN 12 PRODUCTION	2.303	<0.001	0.001
NEUTROPHIL CHEMOTAXIS	2.288	<0.001	0.002
POSITIVE REGULATION OF T CELL PROLIFERATION	2.219	<0.001	0.003
LYMPHOCYTE CHEMOTAXIS	2.208	<0.001	0.003
REGULATION OF B CELL ACTIVATION	2.201	<0.001	0.003
REGULATION OF ANTIGEN PROCESSING AND PRESENTATION	2.196	<0.001	0.003
NADH DEHYDROGENASE COMPLEX ASSEMBLY	2.183	<0.001	0.004
ELECTRON TRANSPORT CHAIN	2.177	<0.001	0.004
NEGATIVE REGULATION OF LEUKOCYTE MEDIATED IMMUNITY	2.175	<0.001	0.004

B. Top 30 gene set enriched in MGMT-L

NAME	NES	NOM p	FDR q
MRNA EXPORT FROM NUCLEUS	-2.286	<0.001	<0.001
SPINDLE ASSEMBLY	-2.267	<0.001	<0.001
HISTONE METHYLATION	-2.263	<0.001	<0.001
RNA EXPORT FROM NUCLEUS	-2.263	<0.001	<0.001
NUCLEAR EXPORT	-2.260	<0.001	<0.001
PEPTIDYL LYSINE METHYLATION	-2.257	<0.001	<0.001
DNA DEPENDENT DNA REPLICATION MAINTENANCE OF FIDELITY	-2.250	<0.001	<0.001
REGULATION OF CHROMOSOME ORGANIZATION	-2.236	<0.001	<0.001
MRNA TRANSPORT	-2.233	<0.001	<0.001
DNA REPLICATION	-2.229	<0.001	<0.001
ESTABLISHMENT OF RNA LOCALIZATION	-2.220	<0.001	<0.001
REPLICATION FORK PROCESSING	-2.220	<0.001	<0.001
REGULATION OF MRNA PROCESSING	-2.209	<0.001	<0.001
MITOTIC SISTER CHROMATID SEGREGATION	-2.205	<0.001	<0.001
CHROMATIN REMODELING	-2.204	<0.001	<0.001
PROTEIN K48 LINKED DEUBIQUITINATION	-2.200	<0.001	<0.001
CHROMATIN ASSEMBLY OR DISASSEMBLY	-2.198	<0.001	<0.001
DNA GEOMETRIC CHANGE	-2.195	<0.001	<0.001
SISTER CHROMATID SEGREGATION	-2.194	<0.001	<0.001
DNA DEPENDENT DNA REPLICATION	-2.190	<0.001	<0.001
PROTEIN LOCALIZATION TO MICROTUBULE ORGANIZING CENTER	-2.190	<0.001	<0.001
REGULATION OF HISTONE METHYLATION	-2.187	<0.001	<0.001
PEPTIDYL LYSINE TRIMETHYLATION	-2.185	<0.001	<0.001
REGULATION OF CHROMOSOME_SEGREGATION	-2.182	<0.001	<0.001
HISTONE H3 K9 METHYLATION	-2.181	<0.001	<0.001
DNA CONFORMATION CHANGE	-2.180	<0.001	<0.001
RNA LOCALIZATION	-2.180	<0.001	<0.001
NEGATIVE REGULATION OF CHROMOSOME ORGANIZATION	-2.179	<0.001	<0.001
COVALENT CHROMATIN MODIFICATION	-2.179	<0.001	<0.001
PROTEIN METHYLATION	-2.179	<0.001	<0.001

NES; normalized enrichment score, NOM p; nominal p-value, FDR q; false discovery rate q-value

Supplementary Table 4. Clinical information, molecular information, analysis results of immunohistochemistry, and results of TIL culture and IFNγ ELISA from the UTH cohort

Patients No.	MGMT-H/L	Age	Sex	Date of surgery	*MGMT* promoter methylation	MGMT expression (FPKM)	MGMT-H/L	CD8 IHC positive area / Tumor area (μm2/mm2)	CD4 IHC positive area / Tumor area (μm2/mm2)	CD20 IHC positive area / Tumor area (μm2/mm2)	CD68 IHC positive area / Tumor area (μm2/mm2)	CD163 IHC positive area / Tumor area (μm2/mm2)	Number of TIL cultured well (wells)	Number of positive TILs proliferation (wells)	TIL culture rate	Number of wells with tumor-reactive immune response (wells)	tumor-reactive immune response rate
3	MGMT-H	83	F	2017/12/27	unmethylated	5.00	H	2.33.E-03	2.59.E-04	3.78.E-05	4.87.E-05	7.37.E-02	24	19	0.792	1	0.042
11	MGMT-H	57	M	2019/12/16	unmethylated	3.72	H	4.23.E-03	1.08.E-03	6.19.E-04	1.88.E-03	5.22.E-01	40	20	0.500	1	0.025
4	MGMT-H	71	M	2018/01/18	unmethylated	3.26	H	3.87.E-04	3.27.E-05	4.78.E-05	3.58.E-05	2.43.E-02	48	17	0.354	3	0.063
2	MGMT-H	65	M	2017/12/14	unmethylated	2.94	H	3.72.E-03	2.58.E-04	9.31.E-05	1.78.E-04	1.23.E-02	24	19	0.792	3	0.125
6	MGMT-H	51	F	2018/08/28	methylated	2.72	H	1.17.E-02	6.09.E-04	5.92.E-04	4.66.E-04	8.17.E-02	24	19	0.792	3	0.125
13	MGMT-H	83	M	2020/11/2	unmethylated	2.30	H	8.20.E-03	1.83.E-03	5.92.E-04	1.96.E-03	4.86.E-01	44	3	0.068	NA	NA
MGMT-H Total													204	97	0.475	11	0.069
7	MGMT-L	36	F	2019/5/20	unmethylated	2.08	L	2.15.E-04	NA	NA	NA	NA	18	0	0	0	0
8	MGMT-L	61	F	2019/5/23	unmethylated	2.03	L	1.58.E-03	1.06.E-04	2.52.E-04	6.93.E-04	6.39.E-02	24	3	0.125	NA	NA
1	MGMT-L	70	F	2017/11/14	methylated	1.45	L	4.18.E-04	2.14.E-04	9.66.E-04	8.78.E-04	2.36.E-01	24	5	0.208	0	0
10	MGMT-L	55	M	2019/10/3	methylated	1.40	L	8.53.E-05	NA	NA	NA	NA	24	17	0.708	1	0.042
12	MGMT-L	78	M	2020/9/15	methylated	1.16	L	4.96.E-04	2.35.E-04	1.65.E-04	1.49.E-04	1.90.E-01	44	0	0	0	0
9	MGMT-L	60	M	2019/6/28	methylated	1.14	L	1.68.E-04	1.07.E-05	9.30.E-06	7.71.E-05	7.67.E-03	21	2	0.095	0	0
5	MGMT-L	53	F	2018/02/06	methylated	0.40	L	3.52.E-04	1.66.E-05	1.54.E-04	5.41.E-05	2.37.E-02	48	26	0.542	0	0
MGMT-L Total													203	53	0.261	1	0.006
Total													407	150	0.369	12	0.035

NA; not available
